# Roles of B Cell-Intrinsic TLR Signals in Systemic Lupus Erythematosus

**DOI:** 10.3390/ijms160613084

**Published:** 2015-06-09

**Authors:** Kongyang Ma, Jingyi Li, Yongfei Fang, Liwei Lu

**Affiliations:** 1 Department of Pathology and Center of Infection and Immunology, Shenzhen Institute of Research and Innovation, The University of Hong Kong, Hong Kong, China; E-Mail: kongyang@hku.hk; 2 Department of Rheumatology, Southwest Hospital, Third Military Medical University, Chongqing 400038, China; E-Mail: lijingyi_tmmu@yahoo.com

**Keywords:** toll-like receptor (TLR), systemic lupus erythematosus (SLE), anti-nuclear autoantibody (ANA), B-1 B cells, transitional B cells, marginal zone B cells (MZ B cells), germinal center B cells (GC B cells), memory B cells, myeloid differentiation primary response gene 88 (MyD88), Unc-93 Homolog B1 (C. elegans) (Unc93b1)

## Abstract

Toll-like receptors (TLRs) are a large family of pattern recognition receptors. TLR signals are involved in the pathogenesis of systemic lupus erythematosus. Mouse and human B cells constitutively express most TLRs. Many B cell subpopulations are highly responsive to certain TLR ligation, including B-1 B cells, transitional B cells, marginal zone B cells, germinal center B cell and memory B cells. The B cell-intrinsic TLR signals play critical roles during lupus process. In this review, roles of B cell-intrinsic TLR2, 4, 7, 8 and 9 signals are discussed during lupus pathogenesis in both mouse model and patients. Moreover, mechanisms underlying TLR ligation-triggered B cell activation and signaling pathways are highlighted.

## 1. Introduction

Systemic lupus erythematosus (SLE) is a systemic autoimmune disease characterized by accumulation of anti-nuclear autoantibodies, hyperactivation of immune cells and multiple organ damages triggered by immune complexes deposition [1,2,3,4]. Overactivation of innate immune response mediated by pattern recognition receptors (PRRs) including toll-like receptors (TLR) is observed in B cells and myeloid cells in various mouse models and patients of SLE [5,6,7,8]. Although TLR signals are not essentially required for B cell activation, B cell-intrinsic TLR signals can drive cell proliferation and differentiation, amplify anti-dsDNA autoantibody and cytokine production [9,10,11,12]. Certain bacteria products and endogenous proteins have been identified to trigger TLR signals. In particular, numerous endogenous products derived from necrotic cells including nucleic acids, high-mobility group protein B1 (HMGB1), heat shock proteins (HSPs) as well as nucleic acid antigen-autoantibody formed immune complexes are involved in B cell overactivation via TLRs and activate T cell-independent antibody response in lupus development [13,14,15]. The endogenous TLR ligands have recently been reviewed [16,17]. For instance, endogenous HMGB1 could trigger TLR2, TLR4 and TLR9 activation whereas endogenous HSP60 and HSP70 only ligate TLR2 and TLR4.

The B cell pool consists of B-1 cells and conventional B-2 cells. Abundant amount of B-1 cells are located in coelomic cavities, but rarely exist in spleen and peripheral lymph nodes. In the peritoneal cavity, B-1 cells are divided into CD5^+^ B-1a and CD5^−^ B-1b subsets with surface CD11b expression. An expanded population of CD11b^+^ B-1 cells has been described in peripheral blood mononuclear cells (PBMCs) of SLE patients [18]. Splenic conventional B-2 cell compartment includes newly formed B cells, immature transitional B cells, mature follicular (FO) B cells, mature marginal zone (MZ) B cells, germinal center (GC) B cells, memory B cells and plasma cells. The abnormal expansion of transitional stage 2 (T2) B cell population is observed in SLE patients [19]. Most available studies on MZ B cells are performed in lupus-mouse models. The populations of autoantibody produced GC B cells and IgG^+^ memory B cells are increased in PBMCs and tonsil of SLE patients [20]. The abnormal responses of B cells, especially the pathogenic role of B cell-derived autoantibodies, have been extensively discussed [21,22]. In particular, TLR activation in B cells can trigger and enhance autoantibody production [23].

This review focuses on recent evidence regarding the involvement of B cell-intrinsic TLR signals during lupus pathogenesis with an emphasis on the contribution of B cell-specific TLR2, 4, 7, 8 and 9 to lupus progression. Current understanding and future perspectives of B cell-intrinsic TLR signals and their downstream key mediators in lupus pathogenesis are also discussed.

## 2. TLR Signal-Sensitive B Cell Subsets in Lupus

**Figure 1:**
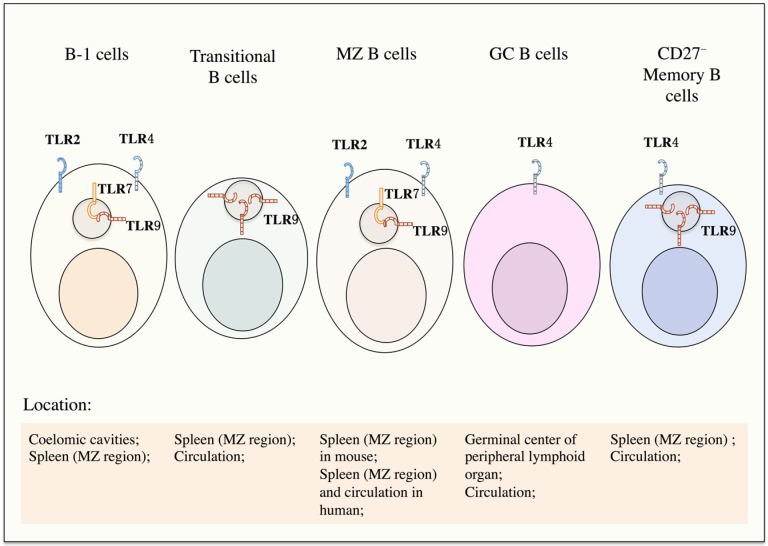
TLR signals in B cell subpopulations. B-1 cells, transitional B cells, marginal zone B cells, germinal center B cells as well as memory B cells are responsive to TLR ligation. B-1 cells are highly sensitive to the ligation of TLR, 2, 4, 7 and 9. CpG ligates the TLR9 in transitional B cells. Marginal zone B cells are more sensitive to the ligation of LPS and CpG. Germinal center B cells respond to TLR4 ligation whereas TLR9 is highly expressed in CD27^−^ memory B cells.

Both B1a and B1b cells from B-1 cells and newly formed B cells, transitional B cells, FO B cells, MZ B cells from conventional B-2 cell pool can recognize TLR ligands, but they show differential responses to TLR ligation. More sensitive TLR signals are found in B-1 cells and MZ B cells. Increased number of B-1 cells is observed in lupus-prone mice and peripheral blood of SLE patients [18,24,25]. Similarly, the enlarged population of MZ B cells is also detected in lupus-prone BXSB mice and markedly contributes to anti-dsDNA autoantibody production [26,27]. Besides B-1 cells and MZ B cells, transitional B cells, germinal center B cells and memory B cells are also highly responsive to certain TLR ligation (Figure 1).

### 2.1. B-1 Cells

B-1 cells, generated from B-1 cell-restricted progenitors with self-renewal property, are characterized with a phenotype of CD19^hi^ CD23^−^ CD43^+^ IgM^hi^ IgD^(variable)^ CD5^±^ [28,29,30]. Peritoneal cavity B-1 cells and B-1 derived plasma cells in bone marrow are considered to be the major source of natural IgM [31,32].

The role of B-1 cells in lupus has been extensively investigated [33]. Upon the elimination of B-1 cells, markedly reduced anti-nuclear autoantibody (ANA) levels with ameliorated lupus nephritis have been detected in lupus-prone NZB/W F1 mice, suggesting that peritoneal B-1 cells may play a pathogenic role during lupus process [34]. Although hypotonic shock for B-1 cell depletion applied in these studies may evoke other cell responses such as serum platelets and epithelia [35,36], the number of T cells and conventional B-2 cells are rarely influenced. Nevertheless, how exactly B-1 cells contribute to lupus pathogenesis still needs further investigation.

B-1 cells are known to express TLRs and respond to TLRs ligation. Surface TLR2, 4 and intracellular TLR7, 9 are highly expressed in mouse B-1 cells [37]. Certain TLR ligands such as CpG1668, LPS, R848 and Pam3CSK4 can selectively promote B-1 cell differentiation [38]. Upon the ligation of TLR4 by LPS, B-1 cells from peritoneal cavity produce a large amount of interleukin-10 (IL10) and inhibit T cell-derived tumor necrosis factor-α (TNFα) secretion [10]. Under the stimulation of CpG, both B1a and B1b cells produce an approximate amount of ANA IgM. Interestingly, compared with B1a cells, CpG-treated B1b cells exhibit a more profound proliferative response with two-fold increase in total cell number. These findings suggest that the major effect of TLR9 signaling is the promotion of antibody secretion in B1a cells but enhancement of cell proliferation in B1b cells.

Recent studies have shown that a key downstream adaptor of TLR9, paired immunoglobulin (Ig)-like receptor B (PIR-B) is activated in B-1 cells upon CpG stimulation. Ligation of TLR9 promotes the phosphorylation of PIR-B in B-1 cells. In Pirb^−/−^ MRL-Fas^lpr/lpr^ mice, accumulated B-1 cells show overactivation of Btk and NF-κb with severer lupus glomerulonephritis [39], indicating a regulatory role of B-1 cell-intrinsic TLR9-PIR-B signaling in lupus mouse model. Furthermore, B1b cells are significantly impaired in lupus-prone FcγRIIB^−/−^TLR9^−/−^ mice, while the number of B1a cell remains comparable. Adoptive transfer of wild type (WT) FcγRIIB^−/−^ B1b cells could significantly reverse the severe lupus nephritis in FcγRIIB^−/−^TLR9^−/−^ mice [40]. These results highlight the different responses towards TLR ligation in B1a and B1b cells during lupus development.

### 2.2. Transitional B Cells

Transitional B cells are immature B-2 cells existing in peripheral blood and secondary lymphoid organs. After migrating into the spleen, transitional B cells differentiate into either follicular B cell (FO B cells) or marginal zone B cell (MZ B cells) [41]. TLR9 is highly expressed in human transitional B cells. Rita Carsetti’s Group shows that ligation of TLR9 with CpG can drive the differentiation of transitional B cells into IgM^+^ memory B cells and natural IgM-secreting plasma cells [42]. Recent studies by Christophe Jamin’s group demonstrate that ligation of TLR9 with CpG could specifically promote human transitional B cell differentiation into ANA-producing “CD24^−^ CD38^+^ CD21^high^ CD23^low^ IgM^high^ IgD^low^ Notch2^high^” MZ B cells, which may play an important role in autoimmune responses [43]. Taken together, these findings have revealed the effect of TLR9 signal on promoting transitional B cell differentiation.

### 2.3. Marginal Zone B Cells

Marginal zone B cells are a distinct non-recirculating B-2 cell population, which are located in the marginal zone (MZ) region of the spleen [44]. In lupus-prone B6.TC mice that express the NZM2410-derived Sle1, Sle2 and Sle3 susceptibility loci, the expanded MZ B cell population is observed. MZ B cells from lupus-prone B6.TC mice show a much stronger response to the ligation of LPS, CpG and chromatin-immune complexes (ICs) with markedly enhanced cell proliferation and production of anti-ssDNA, anti-dsDNA and total IgM [45]. MZ B cells from lupus-prone B6.TC mice produce more IL6 than WT mice whereas levels of MZ B cell-derived IL10 remain comparable [46]. It is known that LPS could promote MZ B cells to secret IL10. However, MZ B cells can secret IL6 upon the treatment of LPS in combination with CD40L [12], indicating the different underlying mechanisms for IL10 and IL6 production by MZ B cells in lupus-prone mice.

Currently, it is unclear whether MZ B cells play a pathogenic role in lupus process. A MZ B cell-derived population with two types of light chains (or more rarely H chains) has been observed in mouse model, which is characterized by autoantibody production and higher sensitivity to TLR7-induced proliferation [47]. In double H chain transgenic mice (3H9H/56R.B6 and MRL/*lpr-Igk^m/h^*), the amplified κ/λ double-positive B cells are identified within the MZ B cell population, which shows the origin of double-positive B cells [48,49,50]. Furthermore, naive B cells from SLE patients show a polyreactive autoantibody response with significantly impaired immunoglobulin κ chain region variation, indicating that the generation of κ/λ double-positive B cells may be due to aberrant processing of early B cell tolerance in lupus [51].

MZ B cells are more responsive to TLR ligation than FO B cells. LPS has been shown to promote the differentiation of MZ B cells but rarely trigger cell differentiation in FO B cells [52]. One of the mechanisms in TLR-mediated B cell activation is that TLR ligation with LPS or CpG could modulate B-cell sensitivity to membrane bounding antigens by formation of F-actin-rich membrane ruffles, a process that is mediated by the activation of cofilin. MZ B cells are much more sensitive to cofilin activation than FO B cells [53]. Besides the *in vitro* sensitivity to TLR ligation in MZ B cells, a marginal zone-restricted CD169^+^ metallophillic macrophage population can capture apoptotic cells and produce CCL22 to induce a regulatory response *in vivo*. By using CCR4 antagonist to block CCL22 for disrupting the location of CD169^+^ metallophillic macrophage, a higher anti-dsDNA IgG level is observed, which indicates that the function of MZ B cells may be modulated by their interaction with tissue-resident macrophages as well [54].

### 2.4. Germinal Center B Cells

It has been reported that the spontaneous germinal center (GC) response is evident in lupus-prone BXD2 mice whereas increased frequency of autoreactive GC B cells exists in lupus patients [20,55,56]. Recent studies have revealed that the differentiation of GC B cells is induced by CD40L, IL21 and IL4 provided from follicular T cells [57,58]. In addition, GC B cells also respond to TLR ligands, especially TLR4 ligands. In spite of the comparable level of TLR4/MD2 surface expression among GC B cells, FO B cells and MZ B cells, GC B cells have as a strong response to LPS-induced proliferation as MZ B cells. Moreover, LPS-stimulated IgM and IgG production is diminished in MyD88^−/−^ B cells, suggesting that the effect of LPS is dependent on MyD88 in GC B cells [52]. In summary, TLR4-MyD88 signal is critically involved in autoreactive GC B cell response.

### 2.5. Memory B Cells

In the peripheral blood of SLE patients, an increased number of CD27^−^ IgD^−^ memory B cells is observed, which is thought to be one major source of ANAs. Memory B cells constitutively express TLR9 [59]. CD27^−^ IgD^−^ memory B cells are highly responsive to CpG-induced cell proliferation. Recently, a novel population of ANA-secreting CD27^−^ Syk^++^ memory B cells has been identified in the peripheral blood of SLE patients, among which 30% are IgD^−^ memory B cells, indicating that they are either two identical populations or overlapped atypical memory B cells. The ligation of TLR4 by LPS has been shown to promote the expansion of CD27^−^ Syk^++^ memory B cells, although underling mechanisms are still unknown [60,61]. Currently, the origin of CD27^−^ memory B cells remains largely unclear. However, CD27^low^ B cells have been found in the marginal zone region of human spleen. Future studies may provide more insight into understanding the role of memory B cells during the development of SLE.

## 3. B Cell-Intrinsic TLR Signals in Lupus

Most TLRs are constitutively expressed in mouse and human B cells [62]. The elevated levels of TLR3, TLR7 and TLR9 in CD19^+^ B cells from PBMCs of SLE patients are detected. In particular, the increased level of TLR9 in B cells is highly correlated with organ damage in lupus patients [63,64]. TLR7 and TLR8 are encoded by X-linked genes, which may render females more susceptible to lupus [65,66]. Differential responses to TLR ligation are observed between humans and mice. Murine B cells are highly sensitive to LPS stimulation via TLR4, while human peripheral blood B cells are not responsive to LPS though tonsil B cells show TLR4 expression [67]. Murine B cells are strongly responsive to TLR2 ligation, but human B cells need BCR activation to trigger TLR2 signal [62]. The extracellular domains of mammalian TLRs from many species have been analyzed by drawing a neighbour-joining molecular tree, which shows that the sequences of human extracellular domains of mammalian TLRs are similar with rat than mice by branch lengths, suggesting the variability of structural and ligation properties between human and mice TLRs [68].

It has been reported that TLR7, TLR8 and TLR9 polymorphisms are associated with the disease incidence of SLE [69,70,71]. Based on recent studies of B cell function in mice lupus models, roles of B cells-intrinsic TLR, 2, 4, 7, 8, 9 signals during lupus pathogenesis are herein discussed (Table 1).

**Table 1:** TLRs in B cells and lupus pathogenesis.

Toll-Like Receptor	Express Pattern on B Cells	Roles of TLR in B Cells	Relevance with SLE Patients	Lupus Pathogenesis in Mouse Model
TLR2	Surface expression on B-1 and MZ B cells.	MZ B cell expansion; B-1 cells differentiation.	Unknown.	LTA (TLR2 ligand)-injection in MRL-Fas^lpr/lpr^mice ↑;
				Pristane-induction in TLR2^−/−^ mice ↓;
				TLR2^−/−^ B6-Fas^lpr/lpr^ mice ↓;
				TLR2^−/−^ MRL-Fas^lpr/lpr^ (N/A).
TLR4	Surface expression on B-1, MZ B, GC B and CD27^−^ Memory B cells.	MZ B proliferation and differentiation;	Accumulated RP105− B cells observed.	LPS (TLR4 ligand)-injection in MRL-Fas^lpr/lpr^ mice ↑; LPS (TLR4 ligand)-injection in NZB/W F1 mice ↑; Pristane-induction in TLR4^−/−^ mice ↓.
		IL10 production;		
		B-1 differentiation;		
		GC B proliferation;		
		CD27^−^ memory		
		B cells proliferation.		
TLR7	Surface and intracellular expression on B-1 and MZ B cells.	T1 B cells and FO B cells expansion;	Increased in B cells; Gene polymorphisms associated with disease.	imiquimod (TLR7 agonist) injection in WT mice ↑;
				R848 (TLR7 agonist) injection in WT mice ↑;
				Transgenic Sle1Tg7 mice ↑;
		IL6 production;		Reconstituted was^−/−^tlr7^−/−^ B cell-μMT mice ↓;
		B-1 cells differentiation.		Reconstituted 3H9 TLR7^−/Yaa^.NZW/ BXSB BM-WT mice ↓.
TLR8	Surface and intracellular expression on B cells.	Controlling TLR7 activation.	Increased in B cells; Gene polymorphisms associated with disease.	TLR8^−/−^ Nba2.Yaa mice ↑;
				TLR8^−/−^ mice ↑;
				TLR8^−/−^ TLR9^−/−^ mice ↑;
				TLR8^−/−^ TLR7^−/−^ mice ↓.
TLR9	Surface and intracellular expression on B-1, MZ B, Transitional B and CD27^−^ Memory B cells.	B1a antibody secretion;	Increased in B ccells; Gene polymorphisms assosiated with disease.	Oligodeoxynucleotides (TLR9 ligand) injection in NZB/W F1 mice ↓;
		B1b proliferation;		
		T2 B cell differentiation;		Reconstituted was^−/−^tlr9^−/−^ B cell-μMT mice ↑;
		MZ B cell proliferation and differentiation;		TLR9^−/−^ MRL-Fas^lpr/lpr^ mice ↑ and TLR9^−/−^ Jh^−/−^ MRL-Fas^lpr/lpr^ mice (N/A);
		CD27^−^ memory B cells proliferation.		
				TLR9^−/−^ CD45E613R BALB/c mice ↓.

↑ accelerated lupus pathogenesis; ↓ ameliorated lupus pathogenesis; (N/A) no difference; MZ marginal zone; GC germinal center.

### 3.1. TLR2

Administration of TLR2 ligand lipoteichoic acid (LTA) into MRL-Fas^lpr/lpr^ mice has been found to promote spontaneous lupus pathogenesis with distinct neuronal dysfunction. Increased IgM^−^ IgG^+^ B220^+^ cells and expansion of CD21^+^ CD23^+^ B cells have been observed in LTA injected MRL-Fas^lpr/lpr^ mice [72]. In pristane-induced lupus mouse model, TLR2^−/−^ mice show lower ANA levels and reduced lupus pathogenesis, together with a reduced level of HMGB1, a well-known endogenous TLR ligand [73]. Moreover, reduced ANA levels, ameliorated glomerulonephritis and decreased expansion of MZ B cells population are also observed in TLR2^−/−^ B6-Fas^lpr/lpr^ mice. However, further studies on MRL-Fas^lpr/lpr^ mice reveal that TLR2 deficiency has no impact on lupus pathogenesis [74]. Based on the pristane-induced lupus model is also performed in B6 background, one possibility for these contradicting findings in TLR2^−/−^ mice is that TLR2 gene polymorphism between B6 and MRL mice strains may contribute to the different outcomes to TLR2 deficiency in lupus development.

### 3.2. TLR4

Several studies have shown that LPS, a TLR4 ligand, can promote lupus nephritis and autoantibody production in lupus-prone MRL-Fas^lpr/lpr^ mice and NZB/W F1 mice [9,75]. Ameliorated autoantibody production and glomerulonephritis are found in TLR4^−/−^ pristane-induced lupus model and lupus-prone TLR4^−/−^ B6. Fas^lpr/lpr^ mice [73,74].

Besides the bacterial product LPS, many endogenous products could also bind to TLR4. The activation of TLR4 signal needs the surface signalosome formation by binding with MD-2 and CD14, while RP105/MD-1 complex can negatively regulate the activation of TLR4/MD2 [76]. It has been shown that surface RP105 directly blocks the TLR4 ligation in cell lines [77,78]. Further analysis of both TLR4/MD2 and RP105/MD-1 reveals a high level of RP105/MD-1 expression in MZ B cells. MZ B cells respond to the ligation of TLR4, RP105 or both, resulting in cell differentiation and antibody production. Activation of RP105/MD-1 has been found to promote TLR4-induced MZ B cell proliferation and survival. However, RP105/MD-1 alone could not drive MZ B cell differentiation [79]. The physical binding with MD-1 is necessary for the function of RP105. Interestingly, increased serum sMD-1 level is observed along with lupus progression in MRL-Fas^lpr/lpr^ mice [80]. In RP105^−/−^ mice, B cell proliferation in response to LPS stimulation is not affected, suggesting a B cell-independent regulation of TLR4 signal by RP105 [81].

BCR, IL6r or CD40 activation through regulating intracellular p-ERK localization could reverse LPS-induced autoantibody production by B cells from 2-12H.B6 mice [82]. In lupus-prone 2-12H. MRL-Fas^lpr/lpr^ mice, the regulatory role of IL6 and CD40 in LPS-induced B cell differentiation is not evident, which might be related with elevated basal p-ERK level in lupus-prone B cells [82]. Besides its interaction with BCR and CD40, TLR4 signal in B cells also has crosstalk with TNFα. DCs and macrophages-derived TNFα could inhibit the LPS-induced B-cell autoantibody production. Notably, LPS could induce severe ANAs production in TNFα^−/−^ mice [83], indicating a complicated crosstalk of TLR4 with BCR, CD40 and TNFα signals in autoreactive B cells.

Moreover, LPS has been considered as an important immune adjuvant, while stimulation of TLR4 could inhibit the antigen-specific immune response through a regulatory role in myeloid cells [84]. As a matter of fact, LPS or apoptotic cells alone could promote IL10 secretion in MZ B cells [12,46], while in MRL-Fas^lpr/lpr^ mice, specific depletion of IL10 in CD19^+^ B cells does not exacerbate lupus nephritis. Evidence suggesting that B cell-derived IL10 may play an insignificant role during lupus progression in lupus-prone MRL-Fas^lpr/lpr^ mice [85].

Despite the lower surface TLR4 level in human B cells, RP105 is highly expressed in naive human B cells. The population of ANAs-secreting RP105^−^ B cell is expended in lupus patients, with the activated B cell phenotype “IgD^−^ CD38^+^ CD95^+^ CD86^+^” [86,87]. Furthermore, accumulated RP105^−^ B lymphocytes with ANAs and IL6 production are also observed in lupus-prone NZB/W F1 mice [88].

### 3.3. TLR7

Many studies in lupus models have indicated a pathogenic role of TLR7 during lupus development. Administration of TLR7 agonists (imiquimod and R848) can induce lupus nephritis with increased serum ANA levels, especially anti-dsDNA IgG levels, in which enhanced activation of autoreactive B cells might be responsible for the autoantibody production [89]. Moreover, blockage of TLR7 or TLR7/9 pathway with immunoregulatory sequences attenuates lupus pathogenesis in MRL-Fas^lpr/lpr^ mice [90].

Recent studies have examined the role of B cell-intrinsic TLR7 by using mouse models with B cell-specific overexpression or deletion of TLR7. In a low-copy TLR7 transgenic B6-Sle1 mouse model (Sle1Tg7), severe autoimmune response and accelerated nephritis have been observed. Further normalization of TLR7 expression in B cells is found to rescue the severe lupus phenotypes in Sle1Tg7 mice [91].

Studies using B cell specific deficiency of Wiskott-Aldrich syndrome protein (WAS) as a lupus mice model also shows significantly elevated ANA levels and glomerulonephritis. By reconstitution of was^−/−^ or was^−/−^tlr7^−/−^B cells in μMT mice, ameliorated lupus pathogenesis has been observed in was^−/−^tlr7^−/−^B cells chimeric mice [92,93]. A recent report using anti-CL/DNA autoantibody 3H9.GFP transgenic mice in lupus-prone NZW/BXSB background has determined the role of TLR7 signal in the differentiation of autoreactive (3H9) B cells. Lower frequencies of GC B cells and plasma cells, together with ameliorated lupus clinical score are observed in the chimeric mice reconstituted with the bone marrow of 3H9 TLR7^−/Yaa^ NZW/BXSB mice in comparison with the bone marrow from 3H9 WT mice, indicating that TLR7 is essential for the spontaneous germinal center response including B cell expansion and diversification in lupus-prone mice [94].

Recent studies have supported the notion that TLR7 is crucial for B cell proliferation and survival [92,93]. The overexpression of TLR7 in TLR7.1Tg mice results in significantly expanded transitional stage 1 B (T1 B) and FO B subpopulations but decreased number of MZ B cells. In WT (Ly5.1^+^) and TLR7.1Tg (Ly5.2^+^) bone marrow reconstituted mice, increased T1 B cell proliferation is specifically observed in TLR7.1Tg B cells, together with increased apoptosis in both T1 and MZ B cells. These data may suggest that an enhanced response of TLR7.1Tg T1 B cells to TLR7 ligation induces cell proliferation and ANA production. Interestingly, the reduced MZ B cell population in TLR7.1Tg mice is dependent on type I IFN signaling [95]. It is clear that TLR7 could be expressed on surface and in intracellular location of B cells, especially in MZ B cells. Upon the internalization of anti-TLR7 antibody binding with surface TLR7, the ligation of TLR7-induced B cells proliferation is largely blocked [96]. However, activating TLR7 by CL095 together with co-activation of BCR can promote IL6 production by B cells, especially in B cells from lupus-prone mice [97].

In human naïve B cells and memory B cells, the ligation of TLR7 by R848 and CpG2006 can promote cell proliferation and IL6 secretion. Additionally, IFNα could amplify the induced IgG and IgM production by R848 in naïve and memory B cells. However, the ligation of TLR2 and TLR4 only shows mild effects on B cell proliferation [98]. Recent reports have demonstrated that B cells from SLE patients have higher activation and necroptosis. Ligation of TLR7 and BCR directly triggers necroptotic B cells formation, which may contribute to B cell lymphopenia in SLE patients [99].

### 3.4. TLR8

TLR8 is one of the X-linked TLRs. The high level of TLR8 in B cells from lupus-prone 564Igi mice is directly related to ANAs production and contributed to the lupus-like pathogenesis in female 564Igi mice [65]. Estrogen treatment has been found to increases the expression level of TLR8 [100]. Notably, overexpression of TLR8 is correlated with local injury in glomerulonephritis [101].

Intriguing findings have been obtained from TLR8 deficient lupus-prone Nba2.Yaa mice, in which accelerated lupus nephritis and splenomegaly is observed, together with enhanced response to TLR7 ligation [102]. Both TLR8^−/−^ and TLR8^−/−^ TLR9^−/−^ mice could develop lupus phenotype by lacking their inhibitory effects on TLR7 signal. In comparison to WT mice, MZ B cells, B1a and B1b cells are impaired in both TLR8^−/−^ and TLR8^−/−^ TLR9^−/−^ mice. The response of TLR8^−/−^ B cells to R848 ligation remains normal, but increased IL6 secretion and CD86 expression in TLR8^−/−^ BM-derived DCs are observed [103]. Ligation of R848 in TLR8^−/−^ TLR9^−/−^ B cells results in the production of large amount of autoantibodies. These findings indicate that cell-specific TLR8 and TLR9 signals are important in regulating lupus development. In conclusion, TLR8 mediates DC-dependent autoantibody production, while TLR9 mediates B cell-intrinsic autoantibody production. Moreover, TLR8^−/−^ TLR7^−/−^ mice show ameliorated lupus pathogenesis as well as restored MZ B cell population [104], indicating a critical function of TLR8 in controlling TLR7 activation [65].

### 3.5. TLR9

Several studies have investigated the role of TLR9 signal in lupus-prone mice. Administration of oligodeoxynucleotides (ODN) with TTAGGG motifs is found to trigger TLR9 activation and block anti-dsDNA autoantibody production in NZB/W F1 mice [105]. Accelerated lupus pathogenesis in TLR9^−/−^ C57 background mice was reported, in which mild ANA production with overactivated TLR7 signal in TLR9^−/−^ B cells is observed [103].

With reconstitution of was^−/−^ or was^−/−^tlr9^−/−^ B cells in μMT mice, severe lupus pathogenesis has been found in was^−/−^tlr9^−/−^ B cells chimeric mice [92,93]. Other studies have examined the chimeric mice with the bone marrow of TLR9^−/−^ MRL-Fas^lpr/lpr^ mice and TLR9^−/−^ Jh^−/−^ MRL-Fas^lpr/lpr^ mice to evaluate the B cell-intrinsic TLR9 signal in lupus process. The population of anti-DNA B cells in the spleen and lymph node is expanded in TLR9^−/−^ MRL-Fas^lpr/lpr^ mice, while the uptake of BrdU is even lower in expanded anti-DNA B cells, suggesting that TLR9 signal restricts the survival of anti-DNA B cells [106]. These findings suggest that B cell-intrinsic TLR9 plays a regulatory role during lupus pathogenesis. However, contradictory findings have been obtained from mice in TLR9^−/−^ BALB/c mice, which do not show ANA production.

Furthermore, a point mutation (CD45E613R) in the autoreactivity-associated locus on chromosome 9 causing constitutively CD45 activation could induce a BALB/c background-dependent ANA production, which is related to TLR9 signal overactivation. By transferring an equal amount of IgH^b^ WT and IgH^a^ CD45E613R bone marrow into WT hosts, all of ANA-secreting cells are generated from CD45E613R mice. Strikingly, in combination with TLR9 deficiency, the ANA production is diminished in CD45E613R mice, indicating a TLR9-dependent autoreactive B cell-intrinsic manner in production of ANAs. The difference of TLR9 signal between BALB/c and C57 genetic backgrounds is probably due to five non-synonymous coding changes located in intracellular toll/IL-1R domain [107]. TLR9^−/−^ B cells highly respond to the stimulation of RNA-associated ICs *in vitro* and *in vivo*, with increased cell proliferation, survival and IgG-secreting cells.

Although comparable TLR7 levels are observed in WT and TLR9^−/−^ B cells, double knock of TLR7 and TLR9 could block the overactivation of TLR9^−/−^ B cells [108]. TLR9-stimulated autoreactive B cell activation is dependent on the binding of the receptor for advanced glycation end products (RAGE) [109]. RAGE deficiency enhances lymphoproliferation with ANA production and lupus nephritis presented in B6-MRL-Fas^lpr/lpr^ mice [110]. This finding could partially explain the regulatory role of TLR9 in lupus process. Moreover, generation of rheumatoid factor (RF) autoreactive B cells is dependent on the ligation of TLR9 [15]. Located at the extra-follicular clusters of both lupus-prone MRL-Fas^lpr/lpr^ mice and B6.Sle1.Sle2.Sle3 (TC) mice, RF B cells can differentiate into RF plasmablasts with the immunization of anti-chromatin IgG2a^a^ ICs through TLR9 dependent pathway [111,112].

TLR9 is expressed in both surface and intracellular region of human B cells. CpG could specifically bind to endosomal TLR9 while anti-TLR9 antibody binds to surface TLR9. Although ligation of endosomal TLR9 with CpG could promote B cells proliferation, the ligation of surface TLR9 with anti-TLR9 antibody blocks both CpG and anti-BCR induced cell proliferation in human B cells [113]. Thus, the molecular mechanisms underlying opposite functions of endosomal and surface TLR9 need to be further investigated.

Available clinical findings show increased percentage of TLR9^+^ B cells in PBMCs from active SLE patients, and the treatment of active SLE serum could increase TLR9 level in B cells [64]. Recent studies observed the reduced protein level and signaling response of TLR9 in B cells from severe SLE patients. Impaired cell proliferation and reduced cytokines (IL6, IL9, IL17A, IFN-γ, MIP-1α and TNF-α) production are observed in CpG triggered B cells from severe SLE patients, suggesting an exhausted status of TLR9 signal in SLE patients [114].

## 4. Key Mediators in B Cell-Intrinsic TLR Signal

Toll/IL-1R (TIR)-domain-containing adaptors including Myeloid Differentiation Primary Response Gene 88 (MyD88), toll-interleukin 1 receptor (TIR) domain containing adaptor protein (TIRAP) and TIR-domain-containing adapter-inducing interferon-β (TRIF), which are essential for transducing the TLR signals. Recent studies have shown that many TLRs share the same downstream adaptor MyD88 except TLR3 [62]. TLR2- and TLR4-mediated signaling pathways are dependent on TIRAP activation [115,116] whereas analog poly(I:C) triggered TLR3 ligation leads to upregulation of TRIF [117].

Internalization of intracellular TLRs including TLR7, TLR8 and TLR9 is dependent on a chaperone protein Unc-93 Homolog B1 (C. elegans) (Unc93b1) [118]. Upon the ligation of TLRs, MyD88 is recruited whereas Unc93b1 is circulated within B cells. Herein, the mechanisms of MyD88 and Unc93b1 in TLR-triggered signaling pathways in B cells are discussed.

### 4.1. MyD88

B cell-intrinsic MyD88 is essential for plasmablast generation, ANA autoantibody secretion in MRL-Fas^lpr/lpr^ mice. CD19-cre mediated MyD88 depletion in B cells ameliorates lupus nephritis in MRL-Fas^lpr/lpr^ mice [119]. MyD88 is responsible for LPS-induced B cell proliferation, cell division and CD86 up-regulation. In contrast, TRIF is indispensable for IL4 and LPS stimulation-induced Aicda expression and μ to γ1 or ε class switch recombination [120]. Based on the protein structure of death domain, MyD88 could bind to several molecules for signal transduction including IFN regulatory factors (IRF4, IRF5 and IRF7) [121,122,123,124]. IRF-5 and IRF-7 mediate the secretion of proinflammatory cytokines and type I interferons (IFNs) by interacting with MyD88. However, IRF4 negatively regulates TLR ligation induced IL6, IL12p40 production by binding to MyD88. IRF4^−/−^ mice are hypersensitive to TLR stimulation [121]. In IRF4 deficiency C57BL/6-lpr/lpr mice, enhanced cytokine production is observed, while lack of plasma cell and reduced autoantibody level leads to ameliorated lupus nephritis [125]. Besides IFN regulatory factors, MyD88 could also bind to single immunoglobulin IL-1R-related protein (SIGIRR). SIGIRR is an inhibitory membrane receptor, which could block TLR4 and TLR9 activation by competitively binding to downstream adaptors as revealed by the findings that SIGIRR^−/−^ splenocytes are highly-responsive to LPS and CpG ligation [126]. Lack of SIGIRR accelerates lupus nephritis in C57BL/6-lpr/lpr mice and hydrocarbon oil-injected mice [127,128].

Lyn deficiency has been shown to induce lupus nephritis, and B cell-specific Lyn deficiency also leads to the development of lupus nephritis [129]. Interestingly, the disease progression of CD19 cre-Lyn^−/−^ mice is similar to Lyn^−/−^ mice. Increased plasma cells, B-1 cells and myeloid cells, together with increased serum IL6 and BAFF levels are observed in CD19 cre-Lyn^−/−^ mice. When combining with the depletion of MyD88 in CD19 cre-Lyn deficient mice, the phenotypes of plasma cell differentiation and lupus nephritis are reversed, suggesting that Lyn play an inhibitory role in MyD88-mediated autoreactive B cell generation in lupus-prone mice [130].

### 4.2. Unc93b1

The internalization and trafficking of nucleic acid-sensing TLRs (including TLR3, 5, 7, 8, 9) are dependent on a chaperone protein Unc93b1 [118,131]. In lupus-prone MRL-Fas^lpr/lpr^ and BXSB background [132], Unc93b1 deficiency can dampen ANA levels and ameliorate lupus nephritis. In WT (IgH^a^):3d (Unc93b1^−/−^) (IgH^b^) reconstituted chimeric mice, ANA secreting cells are generated from WT B cells, demonstrating that Unc93b1 is indispensable for B cell-intrinsic autoantibody production [133].

The D34 site in Unc93b1 is important for its binding to TLRs. TLR9 and TLR7 competitively bind to Unc93B1 at D34 site. Without D34, TLR7 is dominantly binding to Unc93b1 and triggering inflammatory response. In Unc93b1^D34A/D34A^ mice, TLR7-dependent inflammation and B cell-intrinsic TLR7 overactivation are evident. Imiquimod activated TLR7 signal is enhanced in Unc93b1^D34A/D34A^ B cells, while impaired CpG triggered TLR9 signal and comparable lipidA activated TLR4 signal are detected in Unc93b1^D34A/D34A^ B cells [134]. Moreover, levels of surface and interlizated TLR7 are much higher in Unc93b1^D34A/D34A^ MZ B cells. By blockage of TLR7, systemic immune response is blocked in Unc93b1^D34A/D34A^ mice [96]. Further mechanistic studies reveal that Unc93b1 plays different roles in the trafficking of TLR7 and TLR9. Unc93b1 is required in both ER exit and post-Golgi trafficking of TLR9 by binding to AP-2. However, the ER exit process of the intracellular TLR7 trafficking is dependent on Unc93b1 whereas Unc93b1/AP2 complex is not required during this process [135].

## 5. Conclusions and Future Perspectives

B cell-intrinsic TLR signals are crucial for the generation and expansion of autoreactive B cells. This unique signaling process is involved in the pathogenesis of lupus [5]. Since TLRs are also considered as promising therapeutic targets in SLE [136], several biological products have been designed for targeting TLRs, including blocker of TLR7 (IRS661), antagonist of TLR7/8/9 (Chloroquine), inhibitor of TLR2/4 (Vitamin D3). Moreover, antagonist of TLR7/9 (IRS954) and antagonist of TLR7/8/9 (CPG52364) are in clinical phase I stage. In addition, antagonist of TLR7/8/9 (IMO8400) and antagonist of TLR7/9 (IMO3100) are currently in preclinical stage [137].

However, many issues need to be addressed before the clinical application of targeting TLRs. The different effects between human and mouse TLR signals suggest that current mouse models might not be the most appropriate platform for determining the role of TLRs *in vivo*. Thus, it becomes important to constitute the entire human immune system in humanized mice for further investigating the TLR signal. It is equally important to determine whether the blockade of TLR signals may increase the susceptibility to infection. In particular, infection in SLE patients promotes the disease progression by triggering TLR signals [138]. Thus, it might be more important to modulate the hyperactivated TLR signals to the normal level than simply inhibit TLR signals in lupus patients.

Research findings that antibiotics treatment can ameliorate the systemic immune response in TLR4 dependent-lupus prone gp69Tg mice may suggest a pathogenic role of microbiota during lupus development. Thus, the usage of antibiotics might be a promising strategy for combined therapies for SLE. Since there is compelling evidence that a parasitic worm product ES-62 can induce the CD19^+^ CD21^+^ CD23^+^ IL10^+^ regulatory B cell population in MRL-Fas^lpr/lpr^ mice and reverse the lupus pathogenesis [139], further studies are needed to validate the therapeutic potential of regulatory TLR ligands for the treatment of SLE.

## References

[1] Heinlen L.D., McClain M.T., Merrill J., Akbarali Y.W., Edgerton C.C., Harley J.B., James J.A. (2007). Clinical criteria for systemic lupus erythematosus precede diagnosis, and associated autoantibodies are present before clinical symptoms. Arthritis Rheum..

[2] Aronson A.J., Ordonez N.G., Diddie K.R., Ernest J.T. (1979). Immune-complex deposition in the eye in systemic lupus erythematosus. Arch. Int. Med..

[3] Couser W.G., Salant D.J. (1980). *In situ* immune complex formation and glomerular injury. Kidney Int..

[4] Bergtold A., Gavhane A., D’Agati V., Madaio M., Clynes R. (2006). Fcr-bearing myeloid cells are responsible for triggering murine lupus nephritis. J. Immunol..

[5] Meyer-Bahlburg A., Rawlings D.J. (2008). B cell autonomous TLR signaling and autoimmunity. Autoimmun. Rev..

[6] Richez C., Blanco P., Rifkin I., Moreau J.F., Schaeverbeke T. (2011). Role for toll-like receptors in autoimmune disease: The example of systemic lupus erythematosus. Jt. Bone Spine.

[7] Marshak-Rothstein A. (2006). Toll-like receptors in systemic autoimmune disease. Nat. Rev. Immunol..

[8] Patole P.S., Pawar R.D., Lech M., Zecher D., Schmidt H., Segerer S., Ellwart A., Henger A., Kretzler M., Anders H.J. (2006). Expression and regulation of toll-like receptors in lupus-like immune complex glomerulonephritis of MRL-fas(lpr) mice. Nephrol. Dial. Transplant..

[9] Cavallo T., Granholm N.A. (1990). Lipopolysaccharide from gram-negative bacteria enhances polyclonal B cell activation and exacerbates nephritis in MRL/lpr mice. Clin. Exp. Immunol..

[10] Margry B., Kersemakers S.C., Hoek A., Arkesteijn G.J., Wieland W.H., van Eden W., Broere F. (2014). Activated peritoneal cavity B-1a cells possess regulatory B cell properties. PLoS ONE.

[11] Hua Z., Hou B. (2013). TLR signaling in B-cell development and activation. Cell. Mol. Immunol..

[12] Barr T.A., Shen P., Brown S., Lampropoulou V., Roch T., Lawrie S., Fan B., O’Connor R.A., Anderton S.M., Bar-Or A. (2012). B cell depletion therapy ameliorates autoimmune disease through ablation of IL-6-producing B cells. J. Exp. Med..

[13] Rovere-Querini P., Capobianco A., Scaffidi P., Valentinis B., Catalanotti F., Giazzon M., Dumitriu I.E., Muller S., Iannacone M., Traversari C. (2004). Hmgb1 is an endogenous immune adjuvant released by necrotic cells. EMBO Rep..

[14] Barrat F.J., Meeker T., Gregorio J., Chan J.H., Uematsu S., Akira S., Chang B., Duramad O., Coffman R.L. (2005). Nucleic acids of mammalian origin can act as endogenous ligands for toll-like receptors and may promote systemic lupus erythematosus. J. Exp. Med..

[15] Leadbetter E.A., Rifkin I.R., Hohlbaum A.M., Beaudette B.C., Shlomchik M.J., Marshak-Rothstein A. (2002). Chromatin-igg complexes activate B cells by dual engagement of igm and toll-like receptors. Nature.

[16] Yu L., Wang L., Chen S. (2010). Endogenous toll-like receptor ligands and their biological significance. J. Cell. Mol. Med..

[17] Rifkin I.R., Leadbetter E.A., Busconi L., Viglianti G., Marshak-Rothstein A. (2005). Toll-like receptors, endogenous ligands, and systemic autoimmune disease. Immunol. Rev..

[18] Griffin D.O., Rothstein T.L. (2011). A small CD11b^+^ human B1 cell subpopulation stimulates T cells and is expanded in lupus. J. Exp. Med..

[19] Landolt-Marticorena C., Wither R., Reich H., Herzenberg A., Scholey J., Gladman D.D., Urowitz M.B., Fortin P.R., Wither J. (2011). Increased expression of B cell activation factor supports the abnormal expansion of transitional B cells in systemic lupus erythematosus. J. Rheumatol..

[20] Cappione A., Anolik J.H., Pugh-Bernard A., Barnard J., Dutcher P., Silverman G., Sanz I. (2005). Germinal center exclusion of autoreactive B cells is defective in human systemic lupus erythematosus. J. Clin. Investig..

[21] Sang A., Zheng Y.Y., Morel L. (2014). Contributions of B cells to lupus pathogenesis. Mol. Immunol..

[22] Lipsky P.E. (2001). Systemic lupus erythematosus: An autoimmune disease of B cell hyperactivity. Nat. Immunol..

[23] Suurmond J., Diamond B. (2015). Autoantibodies in systemic autoimmune diseases: Specificity and pathogenicity. J. Clin. Investig..

[24] Hayakawa K., Hardy R.R., Parks D.R., Herzenberg L.A. (1983). The “Ly-1 B” cell subpopulation in normal immunodefective, and autoimmune mice. J. Exp. Med..

[25] Qian Y., Conway K.L., Lu X., Seitz H.M., Matsushima G.K., Clarke S.H. (2006). Autoreactive MZ and B-1 B-cell activation by faslpr is coincident with an increased frequency of apoptotic lymphocytes and a defect in macrophage clearance. Blood.

[26] Clark A.G., Fan Q., Brady G.F., Mackin K.M., Coffman E.D., Weston M.L., Foster M.H. (2013). Regulation of basement membrane-reactive B cells in BXSB, (NZBxNZW)F1, NZB, and MRL/lpr lupus mice. Autoimmunity.

[27] Enghard P., Grussie E., Rieder C., Burmester G.R., Riemekasten G. (2011). Subset size, activation threshold and distribution of autoreactive MZ and FO B cells do not differ in a sex-specific manner in the NZB/W F1 murine lupus model: An experimental mouse study. Lupus.

[28] Casola S., Otipoby K.L., Alimzhanov M., Humme S., Uyttersprot N., Kutok J.L., Carroll M.C., Rajewsky K. (2004). B cell receptor signal strength determines B cell fate. Nat. Immunol..

[29] Ghosn E.E., Yang Y., Tung J., Herzenberg L.A., Herzenberg L.A. (2008). CD11b expression distinguishes sequential stages of peritoneal B-1 development. Proc. Natl. Acad. Sci. USA.

[30] Baumgarth N. (2011). The double life of a B-1 cell: Self-reactivity selects for protective effector functions. Nat. Rev. Immunol..

[31] Choi Y.S., Dieter J.A., Rothaeusler K., Luo Z., Baumgarth N. (2012). B-1 cells in the bone marrow are a significant source of natural igm. Eur. J. Immunol..

[32] Reynolds A.E., Kuraoka M., Kelsoe G. (2015). Natural igm is produced by CD5-plasma cells that occupy a distinct survival niche in bone marrow. J. Immunol..

[33] Duan B., Morel L. (2006). Role of B-1a cells in autoimmunity. Autoimmun. Rev..

[34] Murakami M., Yoshioka H., Shirai T., Tsubata T., Honjo T. (1995). Prevention of autoimmune symptoms in autoimmune-prone mice by elimination of b-1 cells. Int. Immunol..

[35] Ubl J., Murer H., Kolb H.A. (1988). Hypotonic shock evokes opening of Ca^2+^-activated K channels in opossum kidney cells. Pflug. Arch..

[36] Kim B.K., Baldini M.G. (1974). The platelet response to hypotonic shock. Its value as an indicator of platelet viability after storage. Transfusion.

[37] Gururajan M., Jacob J., Pulendran B. (2007). Toll-like receptor expression and responsiveness of distinct murine splenic and mucosal B-cell subsets. PLoS ONE.

[38] Genestier L., Taillardet M., Mondiere P., Gheit H., Bella C., Defrance T. (2007). TLR agonists selectively promote terminal plasma cell differentiation of B cell subsets specialized in thymus-independent responses. J. Immunol..

[39] Kubo T., Uchida Y., Watanabe Y., Abe M., Nakamura A., Ono M., Akira S., Takai T. (2009). Augmented TLR9-induced Btk activation in PIR-B-deficient B-1 cells provokes excessive autoantibody production and autoimmunity. J. Exp. Med..

[40] Stoehr A.D., Schoen C.T., Mertes M.M., Eiglmeier S., Holecska V., Lorenz A.K., Schommartz T., Schoen A.L., Hess C., Winkler A. (2011). TLR9 in peritoneal B-1b cells is essential for production of protective self-reactive IgM to control Th17 cells and severe autoimmunity. J. Immunol..

[41] Carsetti R., Rosado M.M., Wardmann H. (2004). Peripheral development of B cells in mouse and man. Immunol. Rev..

[42] Capolunghi F., Cascioli S., Giorda E., Rosado M.M., Plebani A., Auriti C., Seganti G., Zuntini R., Ferrari S., Cagliuso M. (2008). Cpg drives human transitional B cells to terminal differentiation and production of natural antibodies. J. Immunol..

[43] Guerrier T., Youinou P., Pers J.O., Jamin C. (2012). TLR9 drives the development of transitional B cells towards the marginal zone pathway and promotes autoimmunity. J. Autoimmun..

[44] Oliver A.M., Martin F., Gartland G.L., Carter R.H., Kearney J.F. (1997). Marginal zone B cells exhibit unique activation, proliferative and immunoglobulin secretory responses. Eur. J. Immunol..

[45] Zhou Z., Niu H., Zheng Y.Y., Morel L. (2011). Autoreactive marginal zone B cells enter the follicles and interact with CD^4+^ T cells in lupus-prone mice. BMC Immunol..

[46] Sang A., Zheng Y.Y., Yin Y., Dozmorov I., Li H., Hsu H.C., Mountz J.D., Morel L. (2014). Dysregulated cytokine production by dendritic cells modulates B cell responses in the NZM2410 mouse model of lupus. PLoS ONE.

[47] Hehle V., Fraser L.D., Tahir R., Kipling D., Wu Y.C., Lutalo P.M., Cason J., Choong L., D'Cruz D.P., Cope A.P. (2015). Immunoglobulin kappa variable region gene selection during early human B cell development in health and systemic lupus erythematosus. Mol. Immunol..

[48] Yurasov S., Wardemann H., Hammersen J., Tsuiji M., Meffre E., Pascual V., Nussenzweig M.C. (2005). Defective B cell tolerance checkpoints in systemic lupus erythematosus. J. Exp. Med..

[49] Li Y., Li H., Weigert M. (2002). Autoreactive B cells in the marginal zone that express dual receptors. J. Exp. Med..

[50] Fournier E.M., Velez M.G., Leahy K., Swanson C.L., Rubtsov A.V., Torres R.M., Pelanda R. (2012). Dual-reactive B cells are autoreactive and highly enriched in the plasmablast and memory B cell subsets of autoimmune mice. J. Exp. Med..

[51] Giachino C., Padovan E., Lanzavecchia A. (1995). Kappa^+^lambda^+^ dual receptor B cells are present in the human peripheral repertoire. J. Exp. Med..

[52] Meyer-Bahlburg A., Khim S., Rawlings D.J. (2007). B cell intrinsic TLR signals amplify but are not required for humoral immunity. J. Exp. Med..

[53] Freeman S.A., Jaumouille V., Choi K., Hsu B.E., Wong H.S., Abraham L., Graves M.L., Coombs D., Roskelley C.D., Das R. (2015). Toll-like receptor ligands sensitize B-cell receptor signalling by reducing actin-dependent spatial confinement of the receptor. Nat. Commun..

[54] Ravishankar B., Shinde R., Liu H., Chaudhary K., Bradley J., Lemos H.P., Chandler P., Tanaka M., Munn D.H., Mellor A.L. (2014). Marginal zone CD169^+^ macrophages coordinate apoptotic cell driven cellular recruitment and tolerance. Proc. Nat. Acad. Sci. USA.

[55] Grammer A.C., Slota R., Fischer R., Gur H., Girschick H., Yarboro C., Illei G.G., Lipsky P.E. (2003). Abnormal germinal center reactions in systemic lupus erythematosus demonstrated by blockade of cd154-cd40 interactions. J. Clin. Investig..

[56] Arce E., Jackson D.G., Gill M.A., Bennett L.B., Banchereau J., Pascual V. (2001). Increased frequency of pre-germinal center B cells and plasma cell precursors in the blood of children with systemic lupus erythematosus. J. Immunol..

[57] Crotty S. (2011). Follicular helper CD4 T cells (Tfh). Annu. Rev. Immunol..

[58] Basso K., Dalla-Favera R. (2015). Germinal centres and B cell lymphomagenesis. Nat. Rev. Immunol..

[59] Bernasconi N.L., Onai N., Lanzavecchia A. (2003). A role for toll-like receptors in acquired immunity: Up-regulation of TLR9 by bcr triggering in naive B cells and constitutive expression in memory B cells. Blood.

[60] Fleischer S.J., Giesecke C., Mei H.E., Lipsky P.E., Daridon C., Dorner T. (2014). Increased frequency of a unique spleen tyrosine kinase bright memory B cell population in systemic lupus erythematosus. Arthritis Rheumatol..

[61] Wei C., Anolik J., Cappione A., Zheng B., Pugh-Bernard A., Brooks J., Lee E.H., Milner E.C., Sanz I. (2007). A new population of cells lacking expression of cd27 represents a notable component of the B cell memory compartment in systemic lupus erythematosus. J. Immunol..

[62] Bekeredjian-Ding I., Jego G. (2009). Toll-like receptors—Sentries in the B-cell response. Immunology.

[63] Klonowska-Szymczyk A., Wolska A., Robak T., Cebula-Obrzut B., Smolewski P., Robak E. (2014). Expression of toll-like receptors 3, 7, and 9 in peripheral blood mononuclear cells from patients with systemic lupus erythematosus. Med. Inflamm..

[64] Papadimitraki E.D., Choulaki C., Koutala E., Bertsias G., Tsatsanis C., Gergianaki I., Raptopoulou A., Kritikos H.D., Mamalaki C., Sidiropoulos P. (2006). Expansion of toll-like receptor 9-expressing B cells in active systemic lupus erythematosus: Implications for the induction and maintenance of the autoimmune process. Arthritis Rheum..

[65] Umiker B.R., Andersson S., Fernandez L., Korgaokar P., Larbi A., Pilichowska M., Weinkauf C.C., Wortis H.H., Kearney J.F., Imanishi-Kari T. (2014). Dosage of X-linked toll-like receptor 8 determines gender differences in the development of systemic lupus erythematosus. Eur. J. Immunol..

[66] Valle E., Moore S.S., Jann O., Williams J.L., Crews D.H., Benkel B.F. (2005). The bovine cocaine and amphetamine-regulated transcript locus: Gene characterization and snp discovery. Anim. Genet..

[67] Dasari P., Nicholson I.C., Hodge G., Dandie G.W., Zola H. (2005). Expression of toll-like receptors on b lymphocytes. Cell. Immunol..

[68] Werling D., Jann O.C., Offord V., Glass E.J., Coffey T.J. (2009). Variation matters: TLR structure and species-specific pathogen recognition. Trends Immunol..

[69] Wang C.M., Chang S.W., Wu Y.J., Lin J.C., Ho H.H., Chou T.C., Yang B., Wu J., Chen J.Y. (2014). Genetic variations in toll-like receptors (TLRs 3/7/8) are associated with systemic lupus erythematosus in a taiwanese population. Sci. Rep..

[70] Dos Santos B.P., Valverde J.V., Rohr P., Monticielo O.A., Brenol J.C., Xavier R.M., Chies J.A. (2012). TLR7/8/9 polymorphisms and their associations in systemic lupus erythematosus patients from southern brazil. Lupus.

[71] Zhang J., Zhu Q., Meng F., Lei H., Zhao Y. (2014). Association study of TLR-9 polymorphisms and systemic lupus erythematosus in northern chinese han population. Gene.

[72] Dasgupta S., Molano I., Bandyopadhyay M., Kindy M. (2012). Lipoteichoic acid induces B cell activation in female MRL/lpr (fas) mice: Implications for lupus cerebritis. J. Immunol..

[73] Urbonaviciute V., Starke C., Pirschel W., Pohle S., Frey S., Daniel C., Amann K., Schett G., Herrmann M., Voll R.E. (2013). Toll-like receptor 2 is required for autoantibody production and development of renal disease in pristane-induced lupus. Arthritis Rheum..

[74] Freeley S.J., Giorgini A., Tulone C., Popat R.J., Horsfield C., Robson M.G. (2013). Toll-like receptor 2 or toll-like receptor 4 deficiency does not modify lupus in MRLlpr mice. PLoS ONE.

[75] Cavallo T., Granholm N.A. (1990). Bacterial lipopolysaccharide transforms mesangial into proliferative lupus nephritis without interfering with processing of pathogenic immune complexes in NZB/W mice. Am. J. Pathol..

[76] Kimoto M., Nagasawa K., Miyake K. (2003). Role of TLR4/MD-2 and RP105/MD-1 in innate recognition of lipopolysaccharide. Scand. J. Infect. Dis..

[77] Divanovic S., Trompette A., Atabani S.F., Madan R., Golenbock D.T., Visintin A., Finberg R.W., Tarakhovsky A., Vogel S.N., Belkaid Y. (2005). Inhibition of TLR-4/MD-2 signaling by RP105/MD-1. J. Endotoxin Res..

[78] Divanovic S., Trompette A., Atabani S.F., Madan R., Golenbock D.T., Visintin A., Finberg R.W., Tarakhovsky A., Vogel S.N., Belkaid Y. (2005). Negative regulation of toll-like receptor 4 signaling by the toll-like receptor homolog RP105. Nat. Immunol..

[79] Nagai Y., Yanagibashi T., Watanabe Y., Ikutani M., Kariyone A., Ohta S., Hirai Y., Kimoto M., Miyake K., Takatsu K. (2012). The RP105/MD-1 complex is indispensable for TLR4/MD-2-dependent proliferation and igm-secreting plasma cell differentiation of marginal zone B cells. Int. Immunol..

[80] Sasaki S., Nagai Y., Yanagibashi T., Watanabe Y., Ikutani M., Kariyone A., Tsuneyama K., Hirai Y., Takatsu K. (2012). Serum soluble MD-1 levels increase with disease progression in autoimmune prone MRL(lpr/lpr) mice. Mol. Immunol..

[81] Allen J.L., Flick L.M., Divanovic S., Jackson S.W., Bram R., Rawlings D.J., Finkelman F.D., Karp C.L. (2012). Cutting edge: Regulation of TLR4-driven B cell proliferation by RP105 is not B cell autonomous. J. Immunol..

[82] Lee S.R., Rutan J.A., Monteith A.J., Jones S.Z., Kang S.A., Krum K.N., Kilmon M.A., Roques J.R., Wagner N.J., Clarke S.H. (2012). Receptor cross-talk spatially restricts p-ERK during TLR4 stimulation of autoreactive B cells. J. Immunol..

[83] Gilbert M.R., Wagner N.J., Jones S.Z., Wisz A.B., Roques J.R., Krum K.N., Lee S.R., Nickeleit V., Hulbert C., Thomas J.W. (2012). Autoreactive preplasma cells break tolerance in the absence of regulation by dendritic cells and macrophages. J. Immunol..

[84] Rachmawati N.M., Fukudome K., Tsuneyoshi N., Bahrun U., Tsukamoto H., Yanagibashi T., Nagai Y., Takatsu K., Ohta S., Kimoto M. (2013). Inhibition of antibody production *in vivo* by pre-stimulation of toll-like receptor 4 before antigen priming is caused by defective B-cell priming and not impairment in antigen presentation. Int. Immunol..

[85] Teichmann L.L., Kashgarian M., Weaver C.T., Roers A., Muller W., Shlomchik M.J. (2012). B cell derived IL-10 does not regulate spontaneous systemic autoimmunity in MRL.*Fas^lpr^* mice. J. Immunol..

[86] Kikuchi Y., Koarada S., Tada Y., Ushiyama O., Morito F., Suzuki N., Ohta A., Miyake K., Kimoto M., Horiuchi T. (2002). RP105 lacking B cells from lupus patients are responsible for the production of immunoglobulins and autoantibodies. Arthritis Rheum..

[87] Koarada S., Ide M., Haruta Y., Tada Y., Ushiyama O., Morito F., Ohta A., Nagasawa K. (2005). Two cases of antinuclear antibody negative lupus showing increased proportion of B cells lacking RP105. J. Rheumatol..

[88] Fujita K., Akasaka Y., Kuwabara T., Wang B., Tanaka K., Kamata I., Yokoo T., Kinoshita T., Iuchi A., Akishima-Fukasawa Y. (2012). Pathogenesis of lupus-like nephritis through autoimmune antibody produced by cd180-negative b lymphocytes in NZBwf1 mouse. Immunol. Lett..

[89] Yokogawa M., Takaishi M., Nakajima K., Kamijima R., Fujimoto C., Kataoka S., Terada Y., Sano S. (2014). Epicutaneous application of toll-like receptor 7 agonists leads to systemic autoimmunity in wild-type mice: A new model of systemic lupus erythematosus. Arthritis Rheumatol..

[90] Pawar R.D., Ramanjaneyulu A., Kulkarni O.P., Lech M., Segerer S., Anders H.J. (2007). Inhibition of toll-like receptor-7 (TLR-7) or TLR-7 plus TLR-9 attenuates glomerulonephritis and lung injury in experimental lupus. J. Am. Soc. Nephrol..

[91] Hwang S.H., Lee H., Yamamoto M., Jones L.A., Dayalan J., Hopkins R., Zhou X.J., Yarovinsky F., Connolly J.E., Curotto de Lafaille M.A. (2012). B cell TLR7 expression drives anti-rna autoantibody production and exacerbates disease in systemic lupus erythematosus-prone mice. J. Immunol..

[92] Jackson S.W., Scharping N.E., Kolhatkar N.S., Khim S., Schwartz M.A., Li Q.Z., Hudkins K.L., Alpers C.E., Liggitt D., Rawlings D.J. (2014). Opposing impact of B cell-intrinsic TLR7 and TLR9 signals on autoantibody repertoire and systemic inflammation. J. Immunol..

[93] Soni C., Wong E.B., Domeier P.P., Khan T.N., Satoh T., Akira S., Rahman Z.S. (2014). B cell-intrinsic TLR7 signaling is essential for the development of spontaneous germinal centers. J. Immunol..

[94] Boneparth A., Huang W., Bethunaickan R., Woods M., Sahu R., Arora S., Akerman M., Lesser M., Davidson A. (2015). TLR7 influences germinal center selection in murine sle. PLoS ONE.

[95] Giltiay N.V., Chappell C.P., Sun X., Kolhatkar N., Teal T.H., Wiedeman A.E., Kim J., Tanaka L., Buechler M.B., Hamerman J.A. (2013). Overexpression of TLR7 promotes cell-intrinsic expansion and autoantibody production by transitional t1 B cells. J. Exp. Med..

[96] Kanno A., Tanimura N., Ishizaki M., Ohko K., Motoi Y., Onji M., Fukui R., Shimozato T., Yamamoto K., Shibata T. (2015). Targeting cell surface TLR7 for therapeutic intervention in autoimmune diseases. Nat. Commun..

[97] Layer T., Steele A., Goeken J.A., Fleenor S., Lenert P. (2011). Engagement of the B cell receptor for antigen differentially affects B cell responses to toll-like receptor-7 agonists and antagonists in bxsb mice. Clin. Exp. Immunol..

[98] Bekeredjian-Ding I.B., Wagner M., Hornung V., Giese T., Schnurr M., Endres S., Hartmann G. (2005). Plasmacytoid dendritic cells control TLR7 sensitivity of naive B cells via type I IFN. J. Immunol..

[99] Fan H., Liu F., Dong G., Ren D., Xu Y., Dou J., Wang T., Sun L., Hou Y. (2014). Activation-induced necroptosis contributes to B-cell lymphopenia in active systemic lupus erythematosus. Cell Death Dis..

[100] Young N.A., Wu L.C., Burd C.J., Friedman A.K., Kaffenberger B.H., Rajaram M.V., Schlesinger L.S., James H., Shupnik M.A., Jarjour W.N. (2014). Estrogen modulation of endosome-associated toll-like receptor 8: An ifnalpha-independent mechanism of sex-bias in systemic lupus erythematosus. Clin. Immunol..

[101] Kimura J., Ichii O., Miyazono K., Nakamura T., Horino T., Otsuka-Kanazawa S., Kon Y. (2014). Overexpression of toll-like receptor 8 correlates with the progression of podocyte injury in murine autoimmune glomerulonephritis. Sci. Rep..

[102] Tran N.L., Manzin-Lorenzi C., Santiago-Raber M.L. (2014). TLR8 deletion accelerates autoimunity in a mouse model of lupus through a TLR7-dependent mechanism. Immunology.

[103] Desnues B., Macedo A.B., Roussel-Queval A., Bonnardel J., Henri S., Demaria O., Alexopoulou L. (2014). TLR8 on dendritic cells and TLR9 on B cells restrain TLR7-mediated spontaneous autoimmunity in c57bl/6 mice. Proc. Natl. Acad. Sci. USA.

[104] Demaria O., Pagni P.P., Traub S., de Gassart A., Branzk N., Murphy A.J., Valenzuela D.M., Yancopoulos G.D., Flavell R.A., Alexopoulou L. (2010). TLR8 deficiency leads to autoimmunity in mice. J. Clin. Investig..

[105] Dong L., Ito S., Ishii K.J., Klinman D.M. (2005). Suppressive oligodeoxynucleotides delay the onset of glomerulonephritis and prolong survival in lupus-prone NZB x nzw mice. Arthritis Rheum..

[106] Nickerson K.M., Christensen S.R., Cullen J.L., Meng W., Luning Prak E.T., Shlomchik M.J. (2013). TLR9 promotes tolerance by restricting survival of anergic anti-DNA B cells, yet is also required for their activation. J. Immunol..

[107] Mills R.E., Lam V.C., Tan A., Cresalia N., Oksenberg N., Zikherman J., Anderson M., Weiss A., Hermiston M.L. (2015). Unbiased modifier screen reveals that signal strength determines the regulatory role murine TLR9 plays in autoantibody production. J. Immunol..

[108] Nundel K., Green N.M., Shaffer A.L., Moody K.L., Busto P., Eilat D., Miyake K., Oropallo M.A., Cancro M.P., Marshak-Rothstein A. (2015). Cell intrinsic expression of TLR9 in autoreactive B cells constrains BCR/TLR7-dependent responses. J. Immunol..

[109] Tian J., Avalos A.M., Mao S.Y., Chen B., Senthil K., Wu H., Parroche P., Drabic S., Golenbock D., Sirois C. (2007). Toll-like receptor 9-dependent activation by DNA-containing immune complexes is mediated by hmgb1 and rage. Nat. Immunol..

[110] Goury A., Meghraoui-Kheddar A., Belmokhtar K., Vuiblet V., Ortillon J., Jaisson S., Devy J., Le Naour R., Tabary T., Cohen J.H. (2015). Deletion of receptor for advanced glycation end products exacerbates lymphoproliferative syndrome and lupus nephritis in B6-MRL Fas lpr/j mice. J. Immunol..

[111] Herlands R.A., William J., Hershberg U., Shlomchik M.J. (2007). Anti-Chromatin antibodies drive *in vivo* antigen-specific activation and somatic hypermutation of rheumatoid factor B cells at extrafollicular sites. Eur. J. Immunol..

[112] Sang A., Niu H., Cullen J., Choi S.C., Zheng Y.Y., Wang H., Shlomchik M.J., Morel L. (2014). Activation of rheumatoid factor-specific B cells is antigen dependent and occurs preferentially outside of germinal centers in the lupus-prone NZM2410 mouse model. J. Immunol..

[113] Guerrier T., Pochard P., Lahiri A., Youinou P., Pers J.O., Jamin C. (2014). TLR9 expressed on plasma membrane acts as a negative regulator of human B cell response. J. Autoimmun..

[114] Sieber J., Daridon C., Fleischer S.J., Fleischer V., Hiepe F., Alexander T., Heine G., Burmester G.R., Fillatreau S., Dorner T. (2014). Active systemic lupus erythematosus is associated with a reduced cytokine production by B cells in response to TLR9 stimulation. Arthritis Res. Ther..

[115] Yamamoto M., Sato S., Hemmi H., Sanjo H., Uematsu S., Kaisho T., Hoshino K., Takeuchi O., Kobayashi M., Fujita T. (2002). Essential role for tirap in activation of the signalling cascade shared by TLR2 and TLR4. Nature.

[116] Horng T., Barton G.M., Flavell R.A., Medzhitov R. (2002). The adaptor molecule tirap provides signalling specificity for toll-like receptors. Nature.

[117] Yamamoto M., Sato S., Hemmi H., Hoshino K., Kaisho T., Sanjo H., Takeuchi O., Sugiyama M., Okabe M., Takeda K. (2003). Role of adaptor trif in the MyD88-independent toll-like receptor signaling pathway. Science.

[118] Tabeta K., Hoebe K., Janssen E.M., Du X., Georgel P., Crozat K., Mudd S., Mann N., Sovath S., Goode J. (2006). The Unc93b1 mutation 3d disrupts exogenous antigen presentation and signaling via toll-like receptors 3, 7 and 9. Nat. Immunol..

[119] Teichmann L.L., Schenten D., Medzhitov R., Kashgarian M., Shlomchik M.J. (2013). Signals via the adaptor MyD88 in B cells and DCs make distinct and synergistic contributions to immune activation and tissue damage in lupus. Immunity.

[120] Yanagibashi T., Nagai Y., Watanabe Y., Ikutani M., Hirai Y., Takatsu K. (2015). Differential requirements of MyD88 and trif pathways in TLR4-mediated immune responses in murine B cells. Immunol. Lett..

[121] Negishi H., Ohba Y., Yanai H., Takaoka A., Honma K., Yui K., Matsuyama T., Taniguchi T., Honda K. (2005). Negative regulation of toll-like-receptor signaling by IRF-4. Proc. Natl. Acad. Sci. USA.

[122] Kawai T., Sato S., Ishii K.J., Coban C., Hemmi H., Yamamoto M., Terai K., Matsuda M., Inoue J., Uematsu S. (2004). Interferon-alpha induction through toll-like receptors involves a direct interaction of IRF7 with MyD88 and TRAF6. Nat. Immunol..

[123] Honda K., Yanai H., Mizutani T., Negishi H., Shimada N., Suzuki N., Ohba Y., Takaoka A., Yeh W.C., Taniguchi T. (2004). Role of a transductional-transcriptional processor complex involving MyD88 and IRF-7 in toll-like receptor signaling. Proc. Natl. Acad. Sci. USA.

[124] Takaoka A., Yanai H., Kondo S., Duncan G., Negishi H., Mizutani T., Kano S., Honda K., Ohba Y., Mak T.W. (2005). Integral role of IRF-5 in the gene induction programme activated by toll-like receptors. Nature.

[125] Lech M., Weidenbusch M., Kulkarni O.P., Ryu M., Darisipudi M.N., Susanti H.E., Mittruecker H.W., Mak T.W., Anders H.J. (2011). IRF4 deficiency abrogates lupus nephritis despite enhancing systemic cytokine production. J. Am. Soc. Nephrol..

[126] Wald D., Qin J., Zhao Z., Qian Y., Naramura M., Tian L., Towne J., Sims J.E., Stark G.R., Li X. (2003). Sigirr, a negative regulator of toll-like receptor-interleukin 1 receptor signaling. Nat. Immunol..

[127] Lech M., Kulkarni O.P., Pfeiffer S., Savarese E., Krug A., Garlanda C., Mantovani A., Anders H.J. (2008). Tir8/sigirr prevents murine lupus by suppressing the immunostimulatory effects of lupus autoantigens. J. Exp. Med..

[128] Lech M., Skuginna V., Kulkarni O.P., Gong J., Wei T., Stark R.W., Garlanda C., Mantovani A., Anders H.J. (2010). Lack of sigirr/tir8 aggravates hydrocarbon oil-induced lupus nephritis. J. Pathol..

[129] Silver K.L., Crockford T.L., Bouriez-Jones T., Milling S., Lambe T., Cornall R.J. (2007). MyD88-dependent autoimmune disease in lyn-deficient mice. Eur. J. Immunol..

[130] Lamagna C., Hu Y., DeFranco A.L., Lowell C.A. (2014). B cell-specific loss of lyn kinase leads to autoimmunity. J. Immunol..

[131] Itoh H., Tatematsu M., Watanabe A., Iwano K., Funami K., Seya T., Matsumoto M. (2011). Unc93b1 physically associates with human TLR8 and regulates TLR8-mediated signaling. PLoS ONE.

[132] Kono D.H., Haraldsson M.K., Lawson B.R., Pollard K.M., Koh Y.T., Du X., Arnold C.N., Baccala R., Silverman G.J., Beutler B.A. (2009). Endosomal TLR signaling is required for anti-nucleic acid and rheumatoid factor autoantibodies in lupus. Proc. Natl. Acad. Sci. USA.

[133] Koh Y.T., Scatizzi J.C., Gahan J.D., Lawson B.R., Baccala R., Pollard K.M., Beutler B.A., Theofilopoulos A.N., Kono D.H. (2013). Role of nucleic acid-sensing TLRs in diverse autoantibody specificities and anti-nuclear antibody-producing B cells. J. Immunol..

[134] Fukui R., Saitoh S., Kanno A., Onji M., Shibata T., Ito A., Onji M., Matsumoto M., Akira S., Yoshida N. (2011). Unc93b1 restricts systemic lethal inflammation by orchestrating toll-like receptor 7 and 9 trafficking. Immunity.

[135] Lee B.L., Moon J.E., Shu J.H., Yuan L., Newman Z.R., Schekman R., Barton G.M. (2013). Unc93b1 mediates differential trafficking of endosomal TLRs. eLife.

[136] Horton C.G., Pan Z.J., Farris A.D. (2010). Targeting toll-like receptors for treatment of sle. Med. Inflamm..

[137] Connolly D.J., O’Neill L.A. (2012). New developments in toll-like receptor targeted therapeutics. Curr. Opin. Pharmacol..

[138] Sciascia S., Cuadrado M.J., Karim M.Y. (2013). Management of infection in systemic lupus erythematosus. Best Pract. Res. Clin. Rheumatol..

[139] Rodgers D.T., McGrath M.A., Pineda M.A., Al-Riyami L., Rzepecka J., Lumb F., Harnett W., Harnett M.M. (2014). The parasitic worm product, ES-62 targets MyD88-dependent effector mechanisms to suppress ANA production and proteinuria in MRL/lpr mice. Arthritis Rheumatol..

